# Response surface methodology and machine learning optimisations comparisons of recycled AA6061-B_4_C–ZrO_2_ hybrid metal matrix composites via hot forging forming process

**DOI:** 10.1016/j.heliyon.2024.e33138

**Published:** 2024-06-14

**Authors:** Sami Al-Alimi, Nur Kamilah Yusuf, Atef M. Ghaleb, Anbia Adam, Mohd Amri Lajis, Shazarel Shamsudin, Wenbin Zhou, Yahya M. Altharan, yazid saif, Djamal Hissein Didane, Ikhwan S T T, Mohammed Al-fakih, Shehab Abdulhabib Alzaeemi, Abdelghani Bouras, Abdulhafid M A Elfaghi, Haetham G. Mohammed

**Affiliations:** a Sustainable Manufacturing and Recycling Technology (SMART) Research Cluster, Advanced Manufacturing and Materials Centre (AMMC), Universiti Tun Hussein Onn Malaysia (UTHM), 86400, Parit Raja, Batu Pahat, Johor, Malaysia; b Department of Industrial Engineering, College of Engineering, Alfaisal University, 11533, Riyadh, Saudi Arabia; c School of Science and Engineering, University of Dundee, Dundee, DD1 4HN, UK; d Department of Mechanical Engineering, Universiti Teknologi Petronas (UTP), 32610, Bandar Seri Iskandar, Perak, Malaysia; e Faculty of Electrical and Electronic Engineering, Universiti Tun Hussein Onn Malaysia, Parit Raja, 86400, Malaysia; f Sustainable & Responsive Manufacturing Research Group, Fakulti Teknologi dan Kejuruteraan Mekanikal, Universiti Teknikal Malaysia Melaka, 76100, Durian Tunggal, Melaka, Malaysia; g Mathematical Department, Sana'a Community College, Sana'a, Yemen

**Keywords:** Solid-state recycling (SSR), Hot forging (HF), Hybrid materials (HM), Life cycle assessment (LCA), Response surface methodology (RSM), Machine learning (ML)

## Abstract

The optimal conditions of applied factors to reuse Aluminium AA6061 scraps are (450, 500, and 550) ⁰C preheating temperature, (1–15) % Boron Carbide (B_4_C), and Zirconium (ZrO_2_) hybrid reinforced particles at 120 min forging time via Hot Forging (HF) process. The response surface methodology (RSM) and machine learning (ML) were established for the optimisations and comparisons towards materials strength structure. The Ultimate Tensile Strength (UTS) strength and Microhardness (MH) were significantly increased by increasing the processed temperature and reinforced particles because of the material dispersion strengthening. The high melting point of particles caused impedance movements of aluminium ceramics dislocations which need higher plastic deformation force and hence increased the material's mechanical and physical properties. But, beyond Al/10 % B_4_C + 10 % ZrO_2_ the strength and hardness were decreased due to more particle agglomeration distribution. The optimisation tools of both RSM and ML show high agreement between the reported results of applied parameters towards the materials' strength characterisation. The microstructure analysis of Field Emission Scanning Electron Microscopy (FE-SEM) and Atomic Force Microscope (AFM) provides insights mapping behavioural characterisation supports related to strength and hardness properties. The distribution of different volumes of ceramic particle proportion was highlighted. The environmental impacts were also analysed by employing a life cycle assessment (LCA) to identify energy savings because of its fewer processing steps and produce excellent hybrid materials properties.

## 1 Introduction

For years, materials researchers have been creating materials with specific properties required for industrial production. In this study, researchers aim to enhance manufacturing efficiency and cost savings by recycling and reusing materials, while also improving the related forming process by optimizing the processed parameters. Due to economic constraints, production difficulties and materials modification limitations, process enhancement is necessary to achieve further improvement in cost savings, efficiency enhancement, and hybrid material properties. Over the past two decades, composites made from recycled Al-alloys have taken the lead in light metal processing techniques, specifically through solid-state recycling (direct recycling) [1,2] (see Fig. 12).

Solid-state recycling (SSR) forming techniques have gained popularity worldwide due to their ability to simplify the fabrication process. Unlike other forming techniques, SSR does not involve melting processes, making it a competitive and valuable method. In particular, the hot press process has proven to be highly effective in recycling aluminium in composite hybrid forms that display excellent strength and plasticity [[3], [4], [5]].

An investigation was conducted on the hot press method, which is an alternative way of recycling materials. It is similar to the traditional remelting technique. To develop the life cycle assessment model, Simapro 8.0.5 software was used. The Life Cycle Inventory (LCI) data of unprocessed and processed materials used in the background system was obtained from the databases found in the Simapro software. The Ecoinvent database combines information on conventional methods with information published in the literature. The analysis demonstrated that the hot press technique provides significant environmental benefits compared to the standard remelting method. The Global Warming Potential (GWP) value in the hot press method decreased by up to 69.2 % [6]. The hot press revealed viable options for recycling aluminium that was left behind after machining [5,[7], [8], [9], [10]]. An investigation was conducted to compare the hot press method with the traditional remelting technique for recycling materials. The aim was to develop a life cycle assessment model, and Simapro 8.0.5 software was used for this purpose. The unprocessed and processed materials' Life Cycle Inventory (LCI) data used in the background system was obtained from the databases found in the Simapro software. The Ecoinvent database combines information on conventional methods with information published in the literature. The analysis demonstrated that the hot press technique provides significant environmental benefits compared to the standard remelting method. The Global Warming Potential (GWP) value in the hot press method was found to be decreased by up to 69.2 % [[11], [12], [13]]. The hot press method has been found to significantly improve the strength and ductility of recycled aluminium. After undergoing rigorous plastic deformation, recycled aluminium has been shown to possess excellent mechanical and physical properties. Temperature is a crucial factor to consider when working with aluminium alloys, as there is a theoretical linear relationship between temperature, hybrid ceramic particles, and the mechanical characteristics of the alloy. Numerous studies have highlighted the importance of this relationship [[14], [15], [16], [17]].

According to recent literature reviews, there is limited research on hybrid metal matrixes that use nano/micro-sized reinforced particles. However, processing these hybrid materials can improve the technology of forming advanced materials for many applications. For instance, using glass and aluminium-reinforced epoxy can produce a fibre-metal material that serves as a thin interlayer for aircraft. This material consists of layers of glass/fibre epoxy and thin metal (such as aluminum) [18]. B.N.Sarada et al. [19] A study was conducted to investigate the hardness and wear properties of processed LM 25+ Activated Carbon + Mica using stir casting. The results were then compared with those of LM25+ Activated Carbon and LM25+Mica. The researchers concluded that the hybrid form of the material exhibited higher wear properties than those of the materials with single reinforcements. The study was conducted by Aherwar et al. [20] The study investigated the effects of adding waste Porcelain (P) particles (X = 0, 4, 8, 12, and 16) reinforced with B_4_C (4 wt%) and X-wt% reinforced AA7075 aluminium using a stir casting process. Adding particle phases increased the density, microhardness, compressive strength, and tensile strength. Therefore, the AA7075/B_4_C containing 12 wt% Porcelain particulates was the most effective optimum parameter.

To enhance the strength properties of materials, reinforcing B_4_C and ZrO_2_ particles were added to a hybrid recycling process. This process involved solid-state direct recycling and hot press forging to improve the structural properties of the materials. The functional performance of the resulting Metal Matrix Composites (MMCs) was then analysed. To compare this alternative recycling route with the conventional recycling route of Life Cycle Assessment (LCA) and Life Cycle Cost (LCC) using SimaPro 9.2 software. Therefore, the approach for resource efficiency and closed loop circular economy by recycling of manufacturing AA6061 aluminium waste streams to minimize the need for primary material flows and to reduce the related environmental impact. It is believed that this study is considered as one of the economical alternatives that meet the needs of modern societies, by protecting our planet from the adverse consequences of global warming to ensure sustainable consumption and production patterns [21].

Machine learning (ML), as a data-driven scientific research tool, has recently found use in materials science research. Materials such as piezoelectric materials [22], thermoelectric materials [23], stainless steels [24], inorganic materials [25], and high-entropy alloys [26], fall under this category. ML demonstrates its use in creating links between target attributes and experimental variables. While machine learning and artificial intelligence are increasingly being employed in waste modeling [27], additional research into the impact of ML algorithms on the recycling of AA6061 aluminum production waste data is needed.

Machine learning is crucial in materials informatics. ML builds models for specific material properties using material databases, allowing quick prediction of these features. This can speed up the design of new materials and shorten the timescale for material development [28]. Machine learning has played an important role in materials research due to its ability to learn from available data without knowing the underlying physical mechanisms. It has recently emerged as a leading field in materials research [[29], [30], [31], [32], [33]]. Juan et al. [34] have offered a comprehensive analysis of the advances in materials science research that incorporates ML, focusing on its practical applications in metals, batteries, solar materials, and metallic aluminum.

The novel work of forming hybrid material based AA6061 chips reinforced with B_4_C using RSM and ML models to create enhanced mechanical, physical, and microstructural properties. Both optimisation methods have a great agreement towards the compositions of processing parameters for superior hybrid materials performance properties. Therefore, tailoring results for the industrial applications of aircraft implants and automotive resulting high recommendations for sustainability and reusing the materials for the world materials secondary secured materials resources [35]. The limitations of forming hybrid materials include difficulties of achieving dispersion of reinforcements beyond 10 % yield to scaling up the process for investigations on the reinforcements type or size. Further research ought to centre on improving dispersion techniques, taking up manufacturing, employing machine learning for real-time optimisation, developing novel reinforcement exploration approaches, and assessing durability and long-term performance.

## 2 Materials and methods

### 2.1 Experimental material and processing

The Aluminium AA6061 was milled by a Sodick-MC430L machine with a speed cut of v = 110 m/min, feed rate f = 0.05mm/tooth, and cutting depth = 1 mm. To remove impurities a cleaning process with acetone (C_3_H_6_O) is to be conducted following the ASTM G131-96 standards. The next step, the drying process was performed at 90 °C oven temperature for 30 min accordingly. The aluminium AA6061 chips were mixed with B_4_C and ZrO_2_ particles following the mixing theory of hybrid materials and mixed for 2 h by a 3D mixer machine at 35 rpm speed.

The theory of mixing MMC materials relationship is presented to determine the amount of AA6061 chips, and B_4_C and ZrO_2_ contents required for the composite-producing materials as given in Eq (1).

(1) $$\frac{1}{\rho_{C}}=\frac{w_{f}}{\rho_{f}}+\frac{w_{m}}{\rho_{m}}$$

Where *ρ* is density, *w* is volume fraction, while *m*, *f*, and *c* are related to the composites and reinforcement [10,36].

### 2.2 Experimental hot press process

The forging machine could be operated as auto/manual controllers in high pressure up to 47 MPa (35-ton) capacity, four pre-compaction cycles, and 120 min holding time. The process is carried out with (450-500-550) ⁰C temperature above the crystallisation temperature of the recycled samples to form new grains and avoid materials strain hardening by applying constant heat during deformation process. After considering the optimum response by the RSM optimisation software [12,13]. Before testing using a Universal Testing Machine (Shimadzu EHF-EM0100K1-020-0A), the shaping process resulted in the preparation of standard compression specimens (ASTM E9), polishing all the specimens using a Cr_2_O_3_ polishing medium, and etching them in a Keller's and Weck's reagent. Finally, the surface hardness at depth below the surface layer was examined using a Vickers Hardness Tester. While the SU1510 scanning electron microscope (SEM) and an XE-100 from Park Systems, Suwon (AFM) will be used to characterise the microstructures, subsurface layer changes, particle distributions, surface roughness, and grain assessment of the specimens. It is possible to accurately estimate the grain's size and area, which is connected to the features of the nano/micrometric structure. A watershed with a three-level filter works as an auto-statistic count was used to determine the hybrid materials properties [9].

### 2.3 Design of experiment

The optimisation of the hot forming forging processing parameters of the PT, and ceramic volume fraction according to box behnkan design method (BDD), RSM using Minitab 18 software. The three investigated parameters were the volume fraction of added B_4_C, ZrO_2_ particles, and processing temperature that presented in Table 1. Eq (3) presents the RSM general model in a total of eight factorial design points, three replicated centre design points, and four axial design points.

(2) $$Y=b_{0}+\sum_{i=1}^{3}\;b_{i}x_{i}+\sum_{i=1}^{3}bii\;{xi}^{2}+\sum_{i=1}^{2}\;\sum_{i>1}^{3}\;b_{ij}b_{i}b_{j}$$

Where, Y presents the response variable of UTS strength, $b_{0}$ constant value, ε presents the residual error, $b_{i}$ presents the linear value coefficient, $b_{j}$ presents the interaction coefficient and $x_{i}$ presents the coded dimensionless independent variables [9,37]. The R^2^ is near 100 % so, the model would produce good observations and the direct calculations of R^2^ in Eq (3).

(3) $$R^{2}=1‐\;\frac{=\sum_{m=1}^{n}\left(y_{\text{pre},m,m}-t_{\text{mea},m}\right)2}{\sum_{m=1}^{n}\left(t_{\text{mea},m}\right)2}$$

Where *n* is the number of data $y_{pre,m,m},t_{mea,m}$, the predicted and measured values and m data points. The mean absolute error (MAE) is used to calculate the lowest error from the predicted model and expressed in Eq (4) [38].

(4) $$\text{MAE}=\frac{\;y_{pre,m,m}-t_{mea,m}}{n}$$

**Table 1:** Experimental design parameters [37].

Factor	Variation Levels		
	Low (−1)	Medium (0)	High (+1)
Boron Carbide B_4_C (ᵒ%)	5	10	15
Zirconia ZrO_2_ (%)	5	10	15
Processing Temperature (⁰C)	450	500	550

By creating an empirical mathematical relationship using RSM to relate tensile properties with forming variables (operating temperatures and B_4_C and ZrO_2_ hybrid ceramic composite contents, the Desirability Function (DF) will be applied in the optimisation process to generate the contour plot of the feasible region with respect to the responses and parameter constraints. ZrO_2_ contents will be determined by determining the significant factors and the proportion of contribution for each component on the examined responses, and then by forming parameters in touch with formability criteria of recycling hybrid materials recycling.

The benefits of employing factorials, central and axial designs were to determine the optimum parameters of the investigated composites materials and obtain the best tensile strength properties [39]. However, the RSM is obtaining the optimisation and mathematical model directly needed for the problem modelling and analysis that a response of case interests is influenced by several factors [40]. Thus, the aim of optimisation in these findings, the CCD was subjected to the mathematical modelling of tensile strength at a two-factor second-ordered model.

### 2.4 Machine learning algorithms

This study produced waste models that include B_4_C and ZrO_2_ particles to increase the strength properties of materials using a solid-state direct recycling approach and a hot press forging process for AA6061 scrap. The major purpose of these models is to anticipate tensile strength responses resulting from various experiments including factors such as preheating temperature, ZrO_2_, and B_4_C volume fractions. Machine learning was chosen as the modelling technique due to its superior prediction abilities and interpretability of model coefficients, allowing for a better understanding of target parameters and describing the behaviour of real datasets. To this end, the contribution of this paper is the execution of the machine learning model by using Python coding to make the code predictor model. It trains the model to predict the validation data and stores the results. It also prints the best validation error score and displays the predictor. The aim is to discover valuable relationships between attributes within a dataset, which can be harnessed for prediction tasks [26,41].

The steps of the method are as follows.

1 Initialize the data sources by using the following equation:

(5) $$D=\left\{x_{1},x_{2},x_{3},y\right\}$$

where *D* is the set of all data sources, *X*_*1*_ is the Hot Forging (HF), *X*_2_ is the Boron Carbide (B_4_C), *X*_*3*_ is the Zirconium (ZrO_2_), and y is the Actual value for Ultimate Tensile Strength (UTS).

2 Set the respective machine learning model in the following:

(6) $$M=\left\{Machine\;Learning\;byNN\;\left(x_{1},x_{2},x_{3},y\right)\right\}$$

Where M is the set of machine learning model, (x1, x2, x3, y) creates a neural network model with input features x1, x2, and x3, and output feature y [30].

3 Configure hyperparameters for the model:

(7) $$\Theta =\left\{\theta_{1},\theta_{2},\ldots ,\theta_{n}\right\}$$

where *Θ* represent the set of hyperparameters for the ML model, and *θ*1,*θ*2, …,*θn* are the individual hyperparameters that form the set Θ for the ML model.

4 Hyperparameter Optimization [25,27,30]:

(8) $$\Theta^{*}=\mathrm{argmax}=P\left(M\left(X:\theta \right),Y\right)$$

where *M* is the model that takes input features X and predicts output values. *Θ* represents the hyperparameters that guide the learning process of the model *with dataset input X* (HF, B4C, ZrO_2_) and output y (UTS), and *P* is a performance metric.

5 Train machine learning model on each dataset using the selected hyperparameters and make predictions for material properties based on the trained models:

(9) $$Z^{1}=X\;W^{1}+b^{1}$$

(10) $$A^{1}=activation\left(Z^{1}\right)$$

(11) $$Z^{2}=A^{1}\;W^{2}+b^{2}$$

(12) $$A^{2}=activation\left(Z^{2}\right)$$

(13) $$\hat{y}=A^{2}$$

*whereX* is the input matrix with each row representing a data sample (HF, B_4_C, ZrO_2_), *W* represents the weight matrices, *b* represents the bias terms and $\hat{y}$ is the predicted UTS for the given input samples.

6 Evaluate ML output by comparing their outcomes with the original data:

(14) accuracy=compare_outputs(D,ML_outputs)

In the following flowchart in Fig. 2 to show the general workflow of the proposed ML model based on the data.

**Fig. 1:**
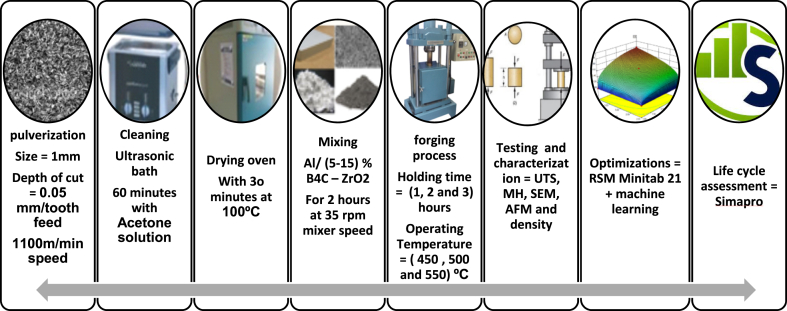
Hot-pressed processing machine and parameters.

**Fig. 2:**
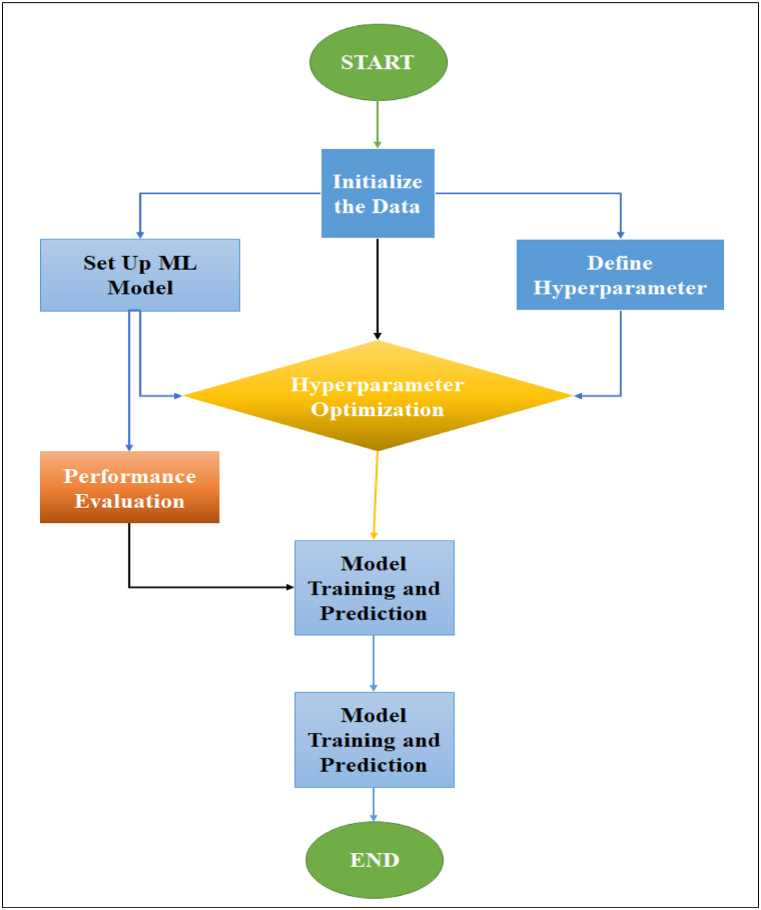
Show the methodology of proposed ML model beside materials recycling technique.

### 2.5 Life cycle assessment methodology and scope definition

**Fig. 3:**
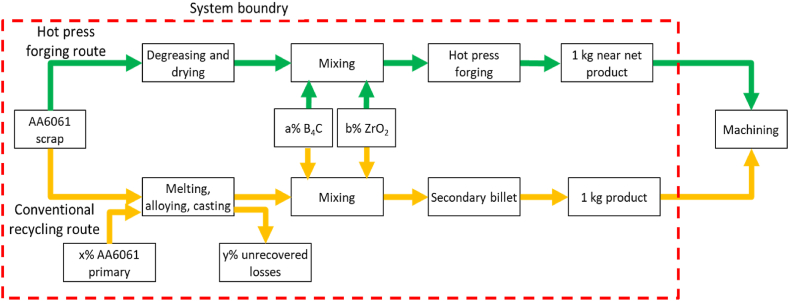
System boundary of AA6061 recycling using hot press forging process.

**Fig. 4:**
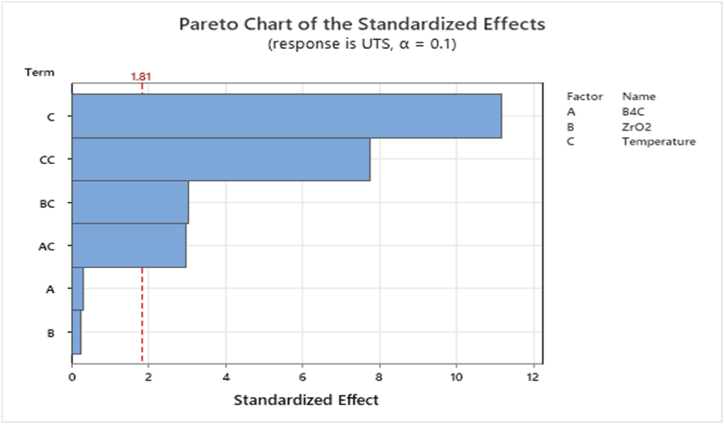
Pareto chart of the standardised effects for the RSM model (B_4_C), (ZrO_2_) and preheated temperature towards UTS strength.

The life cycle assessment model was developed using Simapro 8.0.5 software. The Simapro software provides Life Cycle Inventory (LCI) data for the unprocessed and processed materials used in the background system. The Ecoinvent database is used to compile information on conventional methods. The analysis is used to gather data for the solid-state recycling hot press forging process. The production waste and useful output of the processes, which compress the waste and byproduct streams resulting from manufacturing, form the system boundary of the proposed strategy. The project includes a comparative examination of an alternative material recycling route that starts with the same waste materials as the traditional recycling route. This analysis investigates the technical feasibility of the concept. LCA is a widely used and globally acknowledged process for evaluating a product's impact on the environment. It is a systematic approach to measuring environmental effects and analyzing the interactions that occur with the environment regarding the product or activity under consideration. This part focuses on determining the carbon footprint using global warming potential values for various parameter combinations. The amount of B4C and ZrO_2_ in the hot press forging process is being investigated concerning AA6061 chip recycling. The evaluation methodology and procedural techniques adhere to the ISO14040 and ISO 14044 standards [42,43].

the databases contained inside the SimaPro program provide LCI data for both raw and processed commodities. The data for the conventional technique is collected by utilizing the eco-invent database, which is supplemented with pertinent material derived from scientific publications. As illustrated in Fig. 3, the suggested approach's system boundaries include production waste, useable output from operations, waste compression, and by-product streams that develop during production (see Fig. 1, Fig. 4).

Table 2 provides detailed information on the specific methods, including their key inventory sources and data. The average energy consumption per unit mass of the hot-pressed profile ranges between 25.10 and 53.92 kWh/kg. The ReCiPe approach was used for the life cycle impact assessment (LCIA). The selected strategy. The midpoint indicator for global warming potential (GWP) assessment is a commonly accepted approach for quantifying long-term carbon dioxide equivalent emissions. All emissions are converted into their corresponding 100-year carbon dioxide equivalents using this process [44,45]. The European Aluminium Association's 2008 investigation into gaseous byproducts corresponding to aluminium manufacturing process was condensed into a standard unit of measurement known as carbon dioxide equivalents. Carbon dioxide (CO_2_), methane (CH_4_), sulfur dioxide (SO_2_), nitrous oxide (N_2_O), and perflurocarbon (PFC-14) are five gases that are closely related. CO_2_ gas emissions were prevalent in both routes, however N_2_O and PFC-14 were not found to be important in this process. GWP measurements were used to construct a model to optimize the influence of hot press forging settings on both mechanical property responses through RSM statistical method [46,47].

**Table 2:** Main inventories data and sources for each material.

Material	Details	Source
Aluminium chips AA6061	Aluminium chips from machining milling process	Ecoinvent database v3.1.2022
Boron Carbide (B_4_C)	in fine particles form	Ecoinvent database v3.1.2022
Zirconium (ZrO_2_)	in fine particles form	Ecoinvent database v3.1.2022

### 2.6 Materials and characterisations

The mechanical properties investigated samples in terms of UTS, yield strength, elongation, and hardness. However, UTS tests were conducted with initial strain-rate 2.5 × 10^−3^ s^−1^ at room temperature for all samples that were produced by HF technique following ASTM-E8M standard. Also, hardness tests were conducted with 0.98N–2.94 N with hold time of 10s using Vichers Hardness according to DIN EN ISO 6507–1:2005 standard where, measurements were repeated in several locations for five time of each sample before reading the mean hardness values.

The density is identified as a physical properties test which using Archimedes principle. The density samples were cut into a small pieces of forged hybrid materials following the (Eq) 1. Microstructure of all hybrid's recycled samples were prepared using SiC paper of grits sizes 240, 600 and 1200 each for more than 200 s in each time. Polishing processes were performed with. Finally, the polished samples were electrolytically etched with Barker's reactant method where the voltage = 12V, time = 60s. So, the process permitted the materials structure to be smooth and illuminate the unwanted interferences colours contaminations. Hybrid samples were analysed by Field Emission Scanning Electron Microscopy (FE-SEM), Atomic Force Microscope (AFM) [48].

## 3 Results and discussions

### 3.1 Response surface methodology

Table 3 demonstrates the experimental design and predicted model UTS strength, RSM findings of tensile strength responses were generated after multiple number of experiments which investigated the parameters of preheating temperature, ZrO_2_ and B_4_C volume fraction proportions. The presented pareto chart determines the factors magnitude which are crossing the line reference where, the reference lines confirm that the investigated parameters are significant absolute values cause interactions between parameter towards to the UTS materials strength [2,49,50].

**Table 3:** Analysis of variances.

Source	DF	Adj SS	Adj MS	F-Value	P-Value
Model	6	38393.5	6398.9	48.48	0.000
Linear	3	16504.1	5501.4	41.68	0.000
B_4_C	1	12.5	12.5	0.09	0.765
ZrO_2_	1	8.0	8.0	0.06	0.811
Temperature	1	16483.6	16483.6	124.87	0.000
Square	1	7961.4	7961.4	60.31	0.000
Temperature*Temperature	1	7961.4	7961.4	60.31	0.000
2-Way Interaction	2	2376.4	1188.2	9.00	0.006
B_4_C*Temperature	1	1166.4	1166.4	8.84	0.014
ZrO_2_*Temperature	1	1210.0	1210.0	9.17	0.013
Error	10	1320.0	132.0		
Lack-of-Fit	4	186.0	46.5	0.25	0.902
Pure Error	6	1134.0	189.0		
Total	16	39713.5			

**Fig. 5:**
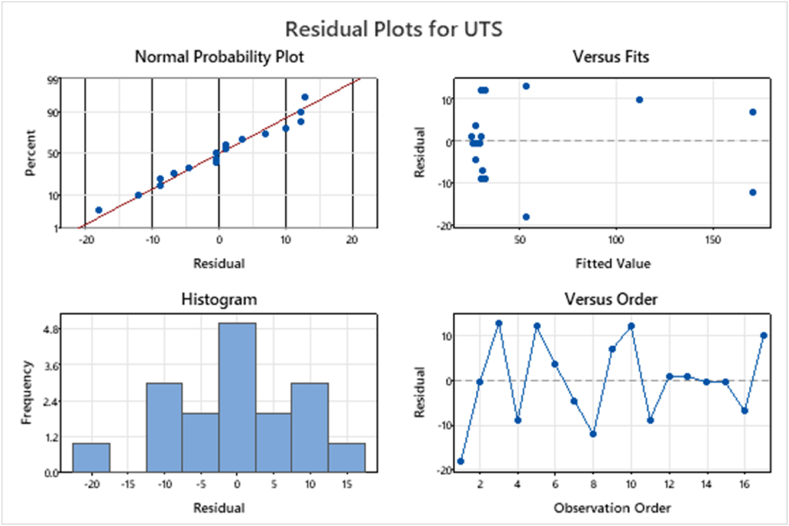
Residual plot for the RSM model (B_4_C), (ZrO_2_) and preheated temperature towards UTS strength.

Fig. 5 depicts UTS's residual plot. In the normal probability plot, the residual for UTS has nearly curvature. The proximity of the graph indicates the errors are negligible since they are still within the acceptable margin. The plot of residuals versus fits response demonstrates the uniformity of variance in an equal distribution, which confirms the approximation of UTS after it is correlated with the RSM [9,51].

**Fig. 6:**
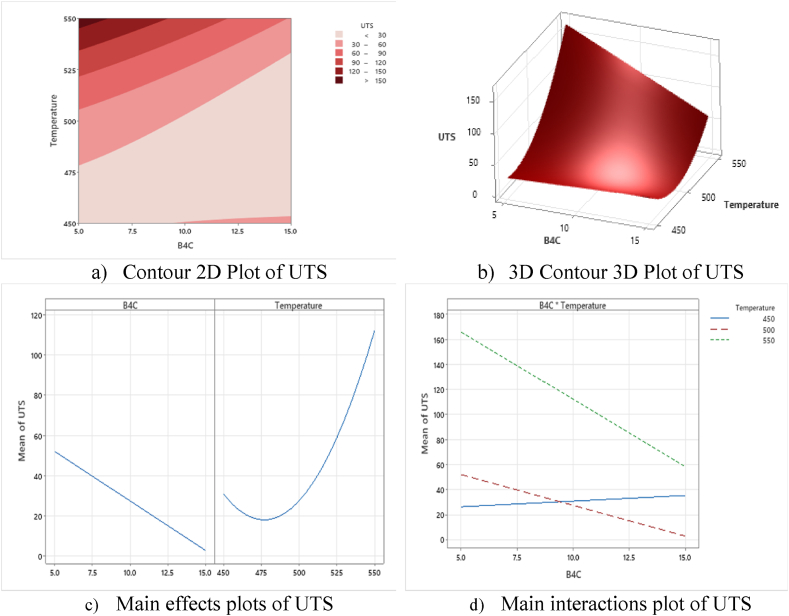
Counters 2D, 3D, and main effects and Interactions plot for the RSM model (B_4_C), (ZrO_2_) and preheated temperature towards UTS strength (a) Contour 2D Plot of UTS, (b) 3D Contour 3D Plot of UTS, (c)Main effects plots of UTS, (d) Main interactions plot of UTS.

The Main effects plots and interactions plots are to identify the most optimised B_4_C, ZrO_2_, and Temperature towards hybrid materials strength MMCs composites, resulting in the most effective and optimal parameters to the composites' strength characteristics. Also, the preheating temperature influences rising UTS according to the major effects plot, the maximum UTS is reached at the highest peak of 550 °C and 5 %. B_4_C– ZrO_2_ reinforced ceramics particles [38,52]. The interplay of temperature and volume fraction impacts the UTS of the materials as shown in the Contour 2D Plot of UTS. The improved UTS ratio includes ideal 5 % B_4_C and ZrO_2_ volume fraction with a 550 °C preheating temperature. But, less than 500 °C and more than 10 % volume proportion of B_4_C and ZrO_2_ had the least influence on the UTS results, as indicated above. The 2D and 3D response surfaces plot shows how preheating temperature and added volume fraction impacts the UTS strength. Fig. 6 shows that UTS grows to its maximum at 5 % volume fraction and 550 °C processing temperature. The typical changes of the investigated experimental factors on the UTS strength response identify the interactions as variations in the design of the reinforced volume percentage of both B_4_C, ZrO_2_ particles and the preheating temperature Increasing the volume fraction and processing temperature in the experimental setup resulted in a more substantial impact on UTS strength up to 5 % vol.B4C, ZrO_2_ and 550 °C While the minimum values of 15 % vol. B_4_C, ZrO_2_ and 450 °C temperature are resulting in continuous declines.

The optimised response parameters settings were reported at 5 % B_4_C, 5 % ZrO_2_ and 550 °C for processing temperatures presented in Table 3.

The data was analysed to determine the optimal level of the investigated factors towards UTS strength qualities, and the regression equation. According to the regression coefficients and analysis of variances of the UTS strength shown in Table 3, the P-value of the preheating temperature is (P-value = 0.000) Also, for B_4_C particles, (P-value = 0.765), ZrO_2_ particles (P-value = 0.811).

Quadratic mathematical modeling is offered to explain the impacts of processing PT temperature B_4_C, and ZrO_2_ volume percent on examined materials' UTS strength response employing the regression model as well as the evaluation of RSM variances. Furthermore, ANOVA shows that the terms A, B, AB, and AA were significant, and the overall model quality may be evaluated using the R-Square, R-Square Adj, prediction R-Square, and Adequate precision values. The quadratic achieved mathematical modeling with a high identification of R-Square = 96 %, which results in an acceptable regression model matched to the research results. Furthermore, the Adj R^2^ and the pred R^2^ are 94 % and 89 %, respectively [10]. The (Eq)14 describes the model equation for the added B_4_C, ZrO_2_ ceramic contents, and processing temperature towards UTS materials strength. The obtained models present the designing parameters experiments to UTS response with the identification of conditions yields to propose the best selected parameters of 5 % B_4_C, 5 % ZrO_2_ and 550 °C and make particular relationship between the applied theories of models and industrials applications. The necessity of strong agreement between the R_2_ Adj and R_2_ pred would avoid overfitting of the mathematical model.

(15) $$\text{UTS}=2938+53.5\;B_{4}C+54.6\;\text{Zr}O_{2}\;‐\;14.60\;\text{Temperature}+0.01759\;\text{Temperature}*\text{Temperature}\;‐\;0.1080\;B_{4}C*\text{Temperature}\;‐\;0.1100\;\text{Zr}O_{2}*\text{Temperature}$$

**Fig. 7:**
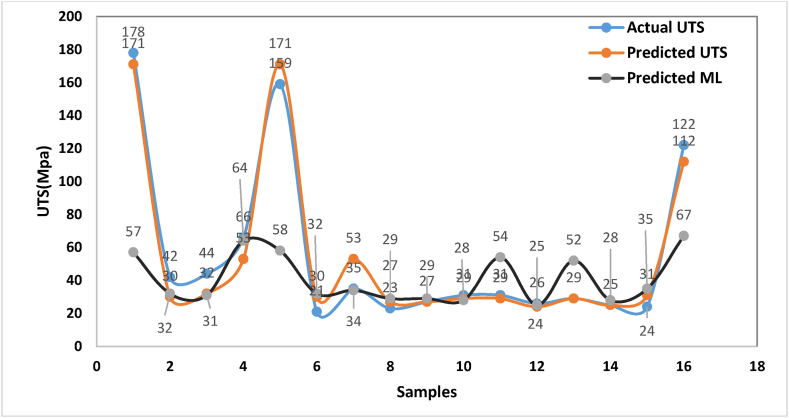
Comparison between actual UTS and predicted UTS.

To confirm the investigations, the comparisons of RSM, ML predicted UTS strength to the actual strength reveal a close match. This indication of both RSM and ML models predict effectively the UTS materials strength and showcase its relationship to the actual estimations and ability of designing, performance accurate predictions precisely, lead to enhance materials processing structural overall usage and efficiencies of applications. Both Table 4 and Fig. 7 show the comparison between the processed experimental parameters of experiments towards UTS response materials structure properties.

**Table 4:** Parametric effects towards materials strength properties.

Samples	B_4_C	ZRO_2_	T (°C)	Actual UTS	Predicted UTS BY (RSM)	Predicted UTS BY (ML)
1	5	5	550	178	171	157
2	15	5	450	42	30	32
3	5	15	450	44	32	31
4	15	15	550	66	53	64
5	5	5	550	159	171	158
6	15	5	450	21	30	32
7	5	15	450	23	32	35
8	15	15	550	35	53	34
9	10	10	500	23	27	26
10	10	10	500	27	27	29
11	10	10	500	31	29	28
12	5	10	500	31	29	54
13	15	10	500	26	24	25
14	10	5	500	29	29	52
15	10	15	500	25	25	28
16	10	10	450	24	31	35
17	10	10	550	122	112	67

### 3.2 Machine learning

The ML model was implemented with a Python program in Microsoft Windows 10, core i7,8th Gen. The proposed model will be evaluated by using four performance metrics Mean Absolute Percentage Error (MAPE), Root Mean Square Error (RMSE), Root relative squared error (RRSE), Accuracy, and coefficient of determination (R^2^). This study chose all these metrics to ensure the prediction task would produce credible results. The equation for each performance metrics are as follows [25,53]:

(16) $$MAPE=100\;*\;\Sigma \left|y-\;\hat{y}\right|\;/\;\Sigma y$$

(17) $$RMSE=\sqrt{\Sigma {\left(y-\;\hat{y}\right)}^{2}/n}$$

(18) $$RRSE=\sqrt{\Sigma {\left(y\;-\;\hat{y}\right)}^{2}\;/\;\Sigma {\left(y\right)}^{2}}$$

(19) $$Accuracy=\frac{Number\;of\;correct\;predictions}{Total\;number\;of\;predictions}\times 100$$

(20) $$R^{2}=1\;-\;\Sigma {\left(y\;-\;\hat{y}\right)}^{2}\;/\;\Sigma {\left(y\;-\;\bar{y}\right)}^{2}$$

**Fig. 8:**
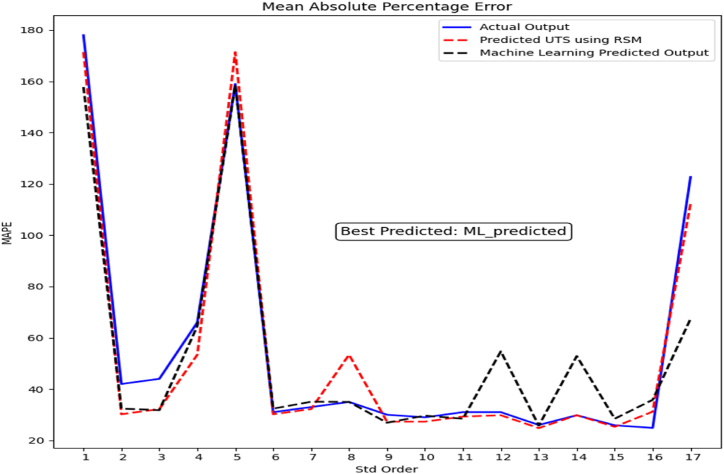
Illustrates the performance evaluation of the ML model using MAPE.

The MAPE findings for proposed machine learning predictions show how accurate the model is at estimating target values. The MAPE is a metric that calculates the average percentage difference between actual and anticipated values. According to Fig. 8, the optimal MAPE value is 0.05 %. A low MAPE, such as 0.05 %, indicates that the model's predictions match the actual values closely, indicating a high level of accuracy. Overall, the MAPE findings of the proposed machine learning model in Fig. 8 illustrate its capacity to make highly correct predictions for most data points. These findings highlight the efficacy of our machine learning approach, as MAPE values near zero suggest that our model successfully captured the fundamental patterns and relationships in the data, making it an excellent candidate for predicting tasks.

**Fig. 9:**
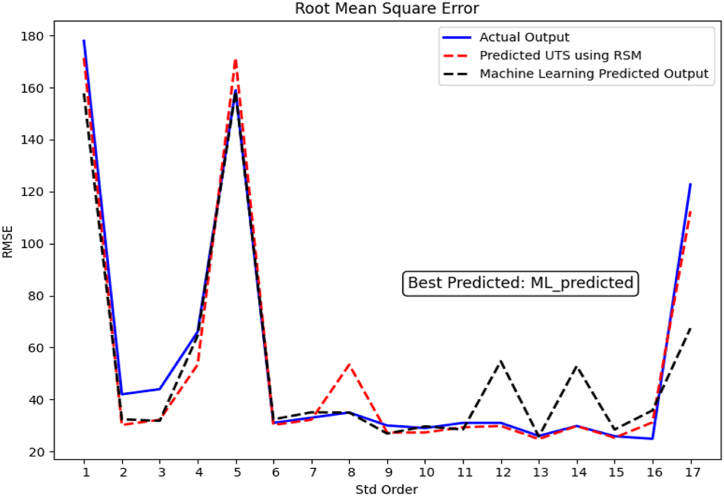
Illustrates the performance evaluation of the ML model using RMSE.

Fig. 9 shows the RMSE value, which achieved outstanding accuracy in the suggested machine learning experiment, with a RMSE as low as 0.02, demonstrating the model's exceptional precision in predicting most data points. Furthermore, the model regularly generated accurate predictions with the lowest RMSE values, showing its excellent ability to match accurate data closely.

**Fig. 10:**
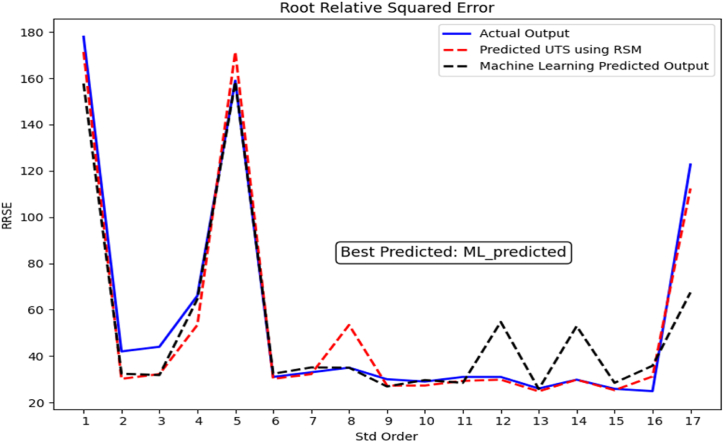
Illustrates the performance evaluation of the ML model using RRSE.

**Fig. 11:**
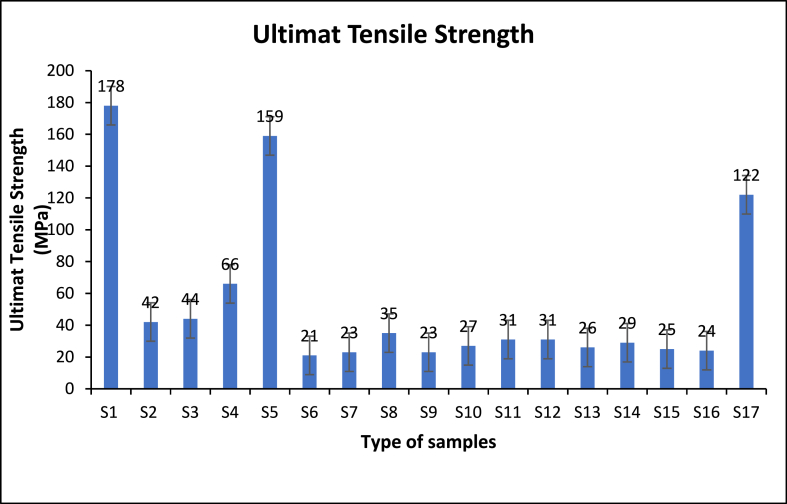
UTS of different processed temperature and B_4_C, ZrO_2_ contents.

In Fig. 10, the Relative Residual Absolute Error (RRAE) metric serves to assess the disparity between predicted values and their corresponding actual values, particularly within the context of HF, B_4_C, ZrO_2_, and the Actual UTS. The insights gleaned from the figure indicate that the model excels in its predictive accuracy when estimating the value of the given data. The model performed admirably, exceeding competing methods in a variety of evaluation metrics. It had the lowest MAPE, RMSE, and RRAE values while achieving a high accuracy of 90.41 %. This exceptional accuracy was observed in estimating that UTS using input data from HF, B_4_C, and ZrO_2_. The model's high correlation coefficient (R^2^) of 0.8699 demonstrates its good predicting abilities for the supplied data. The findings suggest that the created model might be successfully applied in the industrial industry. Using this model, particularly in industries such as reinforcing aluminum AA6061scraps, provides considerable time, cost, and labor efficiency benefits to designers when creating parts with enhanced surface qualities. These proposed models could be helpful decision-making tools for design engineers besides materials recycling techniques (see Fig. 11).

### 3.3 Ultimate tensile strength

The results show that the UTS of the recycled hybrid materials is increased by increasing the amount of added reinforced particles to Al chips. Al/5 % B_4_C + 5 % ZrO_2_ show higher UTS strength compared to other amounts mixing quantities and this value increases to 178 MPa for Al/5 % B_4_C + 5 % ZrO_2_ hybrid materials. Adding reinforced particles causes elastic modulus strength which is attributed to a strong bonding interface between the matrix and the reinforcements [54,55]. Further improvements are because of the interfacial strength dispersion that is achieved under preheating during the forming process. Also, the presence of the hard phase could transfer the load between matrix and reinforcements and increase the total recycled hybrid materials resistance during plastic deformation. The higher thermal mismatch of the aluminium has higher coefficient thermal expansion with the lower reinforced particles during solidification that results in thermal stress generation in the formation of dislocation at the materials interface [56,57]. Furthermore, the increased density dislocations yield to enhance the total strength of recycled hybrid materials. Beyond 5 % B_4_C + 5 % ZrO_2_ the investigated samples were agglomerates; pores are present in the phases the material which is stronger and has more intense stress. and inhomogeneous distributed cause decreases in UTS strength [58].

### 3.4 Micro hardness

The hardness of the hybrid recycled materials is evaluated by applying Vicker hardness tester and the dwell time and applied load are 3 kg and 15s respectively. An average of five reading is reported from each hardness value. Where according to Eqs. (20), (21) [55].

(21) λ=4(1−f)r/3f

Where, *λ* is the distance of reinforced particles, *r* is the radius of ceramic particles, and *f* is the volume fraction of ceramic particles. The findings show that, low ratio of reinforcements was significant when the microhardness is in concern. Increasing the volume fraction of the hybrid particles cause increases in the total materials hardness due to the incorporation of particles with the matrix and the presence of relative hard particles. It is noted that Al/5 % B_4_C + 5 % ZrO_2_ is 42 % greater than Al/15 % B_4_C + 5 % ZrO_2_ and 40 % higher than Al/10 % B_4_C + 10 % ZrO_2_. By increasing the reinforced volume fraction will cause decreases in the distance between added particles due to the materials agglomeration and pores presence with materials discontinuity phase of the material which is greater and higher stress. So, the relationship Eq. (k) illustrates effects of continuous added reinforcements [59]:

(22) $${\;\tau }_{⁰}=\left(Gb/\lambda \right)$$

Where, ${\;\tau }_{⁰}$ shearing stress, shearing module, *b* is the Burger's crystal vector and *λ* is the distance of reinforced particles [59]. The reinforcements volume fraction with low ratio are more significant to improve hardness quality. More than 5 %, the hardness-strength would be declined and the materials will be favourable for composites machinability [57].

### 3.5 Density

Generally, ceramics particles have significant effects on total composites densities. The additions of lightweight's reinforcements such as B_4_C cause densities reductions of the hybrid composites materials unlike ZrO_2_ reinforcements which cause addition of the hybrid composites. Figure show that the hybrid composites densities of Al/15 % B_4_C + 15 % ZrO_2_ is 2.78 kg/m^3^ which higher of all investigated mixed samples Al - 5 % B_4_C + 5 % ZrO_2_ and 10 % B_4_C + 10 % ZrO_2_. The increases in the hybrid materials' densities indicate the breakage may not influence the hybrid materials' interfacial bonds between the particles and the matrix [60,61]. The density of the examined samples drops, which may be attributed to the lower overall densities of B_4_C particles compared to their densities of pure AA6061 chips and ZrO_2_. The density of composites made of metal matrix is contingent upon the mixed percentage of materials with respect to the AA6061 chips. The study utilised the mixing rule theory to determine the densities of composite samples. According to the principles of the mixing rule, it is seen that when the proportion of reinforced ceramic added contents exceeds 10 %, certain effects are observed [39].

### 3.6 Field Emission Scanning Electron Microscopy (FE-SEM) and Atomic Force Microscope (AFM)

The recycled hybrid materials based AA6061 chips reinforced with B_4_C and ZrO_2_ contents of different proportions were investigated by means FE-SEM/AFM (see Fig. 13).

Atomic Force Microscope (AFM) is widely recognised as the prevailing technique for investigating and quantifying surface roughness, as well as examining the morphology of hybrid composites samples surfaces with micron to nanoparticle dimensions. Nevertheless, hybrid composites samples possess a polycrystalline structure and grain size, rendering them suitable for research endeavours in hybrid composites samples technology. Additionally, the form of an object is a determining factor that impacts the dimensions of its grain, such as radius length and areas. Consequently, it has been found that the utilisation of a sharp tip leads to increased accuracy in accordance with the research conducted by Rifai et al. [62]. The findings indicate the potential for mitigating particle agglomeration along the samples. However, the outcomes were modified with the reduced grain sizes are documented in Table 5, illustrating a decrease in size from the lowest mixing proportions of reinforced contents as indicated. According to Attila bony et al [63]. There is a reduction in reliance on the surface roughness of pictures for conducting testing in Fig. 14. The impacts of process on the form, features, surface structure, and descriptive responses of materials thermal forming process leads to surfaces that are softer and more malleable.

**Table 5:** Mean roughness parameters of AA6061 chips reinforced hybrid materials.

no	Samples	Area (μm^2^)	Length (μm^3^)	Peri (μm^3^)	Rpv (nm)	Rq (nm)	Ra (nm)
1	Al/5 % B_4_C + 5 % ZrO_2_	0.040	0.27	0.85	33.06	6.99	5.53
2	Al/15 % B_4_C + 5 % ZrO_2_	0.020	0.21	0.67	61.37	13.66	10.85
3	Al/5 % B_4_C + 15 % ZrO_2_	0.027	0.237	0.72	69.35	14.91	11.73
4	Al/10 % B_4_C + 10 % ZrO_2_	0.028	0.239	0.73	52.56	11.26	8.86
5	Al/15 % B_4_C + 15 % ZrO_2_	0.047	0.305	0.93	40.56	8.93	6.59

**Fig. 12:**
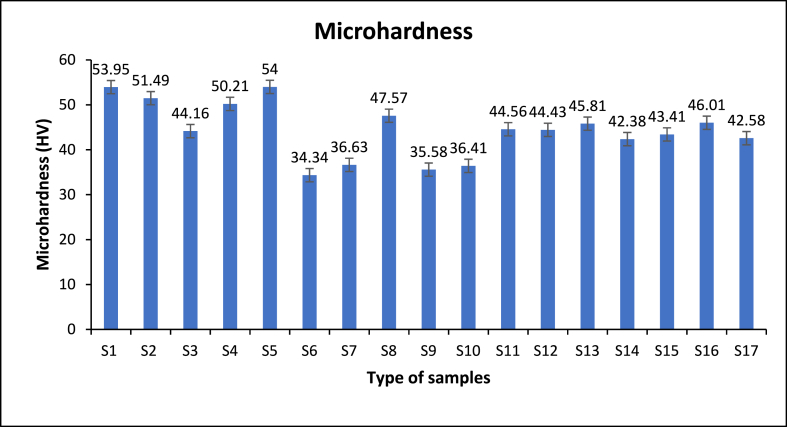
Microhardness of different processed temperature and B_4_C, ZrO_2_ contents.

**Fig. 13:**
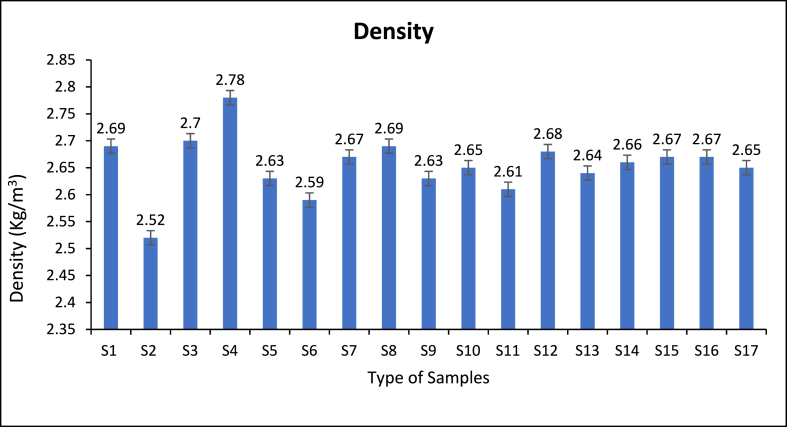
Density of different processed temperature and B_4_C, ZrO_2_ contents.

**Fig. 14:**
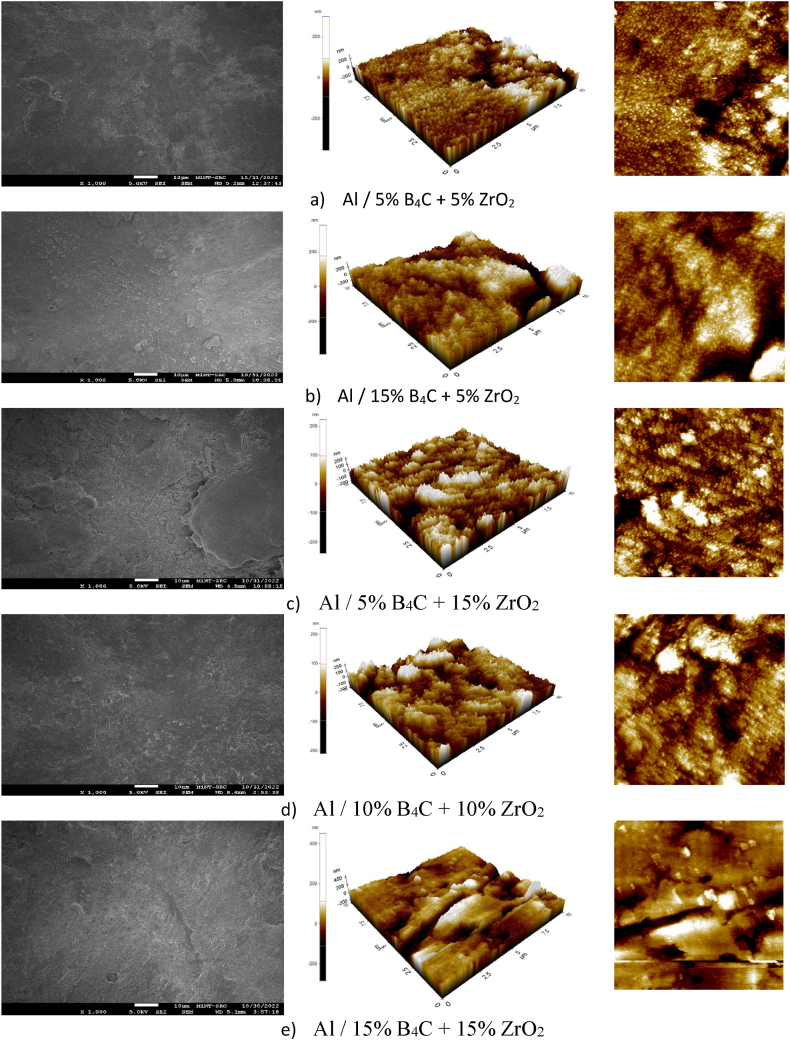
FE-SEM/AFM AA6061 chips reinforced B4C/ZRO_2_ hybrid materials Al/(a) 5 % B_4_C + 5 % ZrO_2_, (b) Al/15 % B_4_C + 5 % ZrO_2_, (c) Al/5 % B_4_C + 15 % ZrO_2_, (d) Al/10 % B_4_C + 10 % ZrO_2_, and (e) Al/15 % B_4_C + 15 % ZrO_2_.

### 3.7 Life cycle assessment

**Fig. 15:**
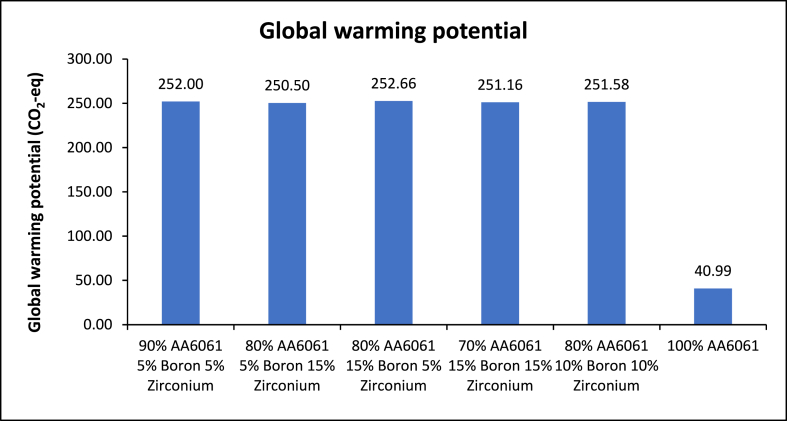
Global warming potential of the different content of B_4_C and ZrO_2_.

**Fig. 16:**
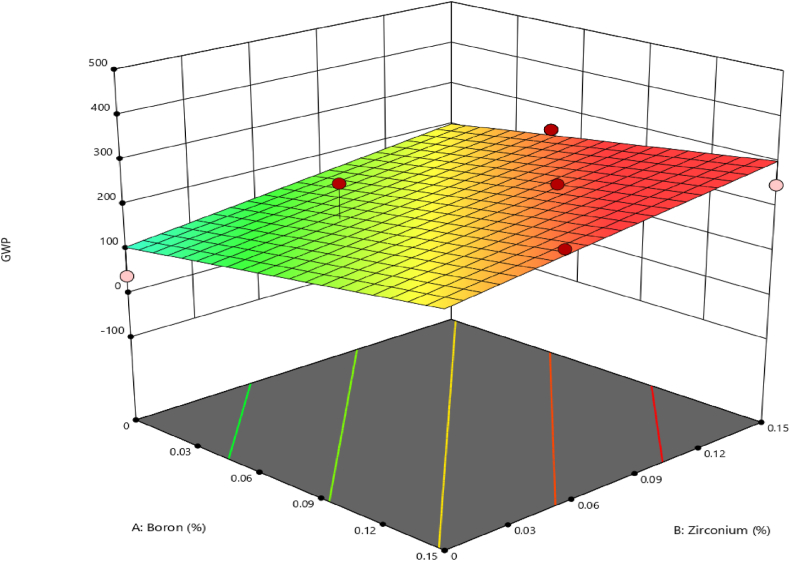
3D surface graphs for GWP with the relationship to B_4_C and ZrO_2_ ceramics contents.

Fig. 15 depicts the results of the global warming potential of various B_4_C and ZrO_2_ concentrations. The results show that the varied compositions result in somewhat different GWP levels. The composition of 80 % Aluminium +15 % B_4_C + 5 % ZrO_2_ has the greatest GWP of 252.66 CO_2_-eq, whereas the composition of 80 % Aluminium +5 % B_4_C + 15 % ZrO_2_ has the lowest GWP of 250.5 CO_2_-eq. These discrepancies might be attributable to varying B_4_C and ZrO_2_ proportions, demonstrating that modest changes in alloy composition can influence its environmental effect in terms of GWP (see Fig. 16).

The inclusion of boron carbide raises the GWP value for a variety of reasons, most notably its raw material manufacturing procedure. Boron carbide is commonly synthesised from boron oxide and carbon by a high-temperature reduction method that is energy-intensive [64]. Furthermore, the manufacturing of boron carbide necessitates energy-intensive methods such as high-temperature carbothermal reduction of boron oxide. Its manufacturing involves high temperatures and complicated chemical processes, which might lead to a larger carbon footprint. whereas carbon the carbon footprint of boron carbide raw materials is influenced by their intensity. Boron carbide has a relatively high carbon content, therefore the mining and processing of carbon can have a considerable carbon impact [65].

While the inclusion of ZrO_2_ increases the GWP value of the recycling process, this is mostly since ZrO_2_ manufacture frequently includes energy-intensive procedures, notably the reduction of ZrO_2_ tetrachloride using magnesium in the Kroll process [66]. This high-temperature reduction process consumes a lot of energy, which contributes to a bigger carbon footprint. Furthermore, ZrO_2_ is a more chemically complicated element, and its extraction and purification can need many stages, each of which requires energy and resources. The intricacy of the relevant chemical processes might lead to a larger carbon footprint. Furthermore, the extraction of ZrO_2_ raw materials, such as zircon sand, may include energy-intensive mining operations and extraction procedures, adding to the total carbon footprint [67]. It was stated that the regression model significance of the test for significance on the particularised coefficients model and the test for lack-of-fit should be carried out. The results of the tests were typically summarised using an ANOVA. After pooling, the final quadratic model equation in terms of coded elements for GWP is as follows in the case of coded factors [68].

(23) GWP=205.54+50.05A+48.43B

Where, *A* is the percentage of B_4_C while *B* is the percentage of ZrO_2_ contents. The 3D surface graphs for GWP are shown in Fig. 15 the GWP tends to increase considerably with increases of the B_4_C and ZrO_2_ contents. Hence, maximum GWP is obtained.

## 4 Conclusion

Hybrid MMCs recycling composites up to 15 % for B_4_C and ZrO_2_ particles reinforced aluminium AA6061 scraps were fabricated by using HF technique. The UTS results of the investigated materials were optimised and compared by employing RSM and ML methods. From the microstructure findings, uniform distribution of B_4_C and ZrO_2_ particles in the matrix for samples up to 10 % reinforced particles contents and beyond the optimised value the agglomerations of particles would be observed. Due to that, the effects of adding reinforcing particles cause UTS strength increase because of increase in the dislocation density beyond 5 % reinforced particles concentration, the UTS and MH would be decreased. While the density is relative to the total added reinforced particles. The LCA outcomes are given in utilitarian units, which evaluate the environmental effect per kilogram of aluminium compound and reinforced particles. The established approach to determine the midpoint indicator that GWP was used to quantify CO_2_ equivalent emissions towards time. Within 100 years. The study presents the materials characterisations investigations of the hybrid recycled materials and optimisation by employing RSM and ML to propose the materials for the automotives manufacturing applications.

## Data availability statement

All generated data or analysed during this study are included in this published article.

## CRediT authorship contribution statement

**Sami Al-Alimi:** Writing – original draft, Validation, Methodology, Formal analysis. **Nur Kamilah Yusuf:** Supervision, Project administration. **Atef M. Ghaleb:** Resources, Funding acquisition. **Anbia Adam:** Validation, Funding acquisition, Conceptualization. **Mohd Amri Lajis:** Visualization. **Shazarel Shamsudin:** Investigation. **Wenbin Zhou:** Writing – review & editing. **Yahya M. Altharan:** Resources. **yazid saif:** Investigation. **Djamal Hissein Didane:** Validation. **Ikhwan S T T:** Data curation. **Mohammed Al-fakih:** Data curation. **Shehab Abdulhabib Alzaeemi:** Validation. **Abdelghani Bouras:** Conceptualization. **Abdulhafid M A Elfaghi:** Investigation. **Haetham G. Mohammed:** Conceptualization.

## Declaration of competing interest

The authors declare that they have no known competing financial interests or personal relationships that could have appeared to influence the work reported in this paper.
